# MIP-3α-antigen fusion DNA vaccine enhances sex differences in tuberculosis model and alters dendritic cell activity early post vaccination

**DOI:** 10.21203/rs.3.rs-5663995/v1

**Published:** 2025-01-14

**Authors:** James T. Gordy, Rowan E. Bates, Elizabeth Glass, Jacob Meza, Yangchen Li, Courtney Schill, Alannah D. Taylor, Tianyin Wang, Fengyixin Chen, Khaleel Plunkett, Styliani Karanika, Petros C. Karakousis, Richard B. Markham

**Affiliations:** Johns Hopkins School of Public Health; Johns Hopkins School of Public Health; Johns Hopkins School of Public Health; Johns Hopkins School of Public Health; Johns Hopkins School of Public Health; Johns Hopkins School of Public Health; Johns Hopkins School of Public Health; Johns Hopkins School of Public Health; Johns Hopkins School of Public Health; Johns Hopkins School of Public Health; Johns Hopkins University School of Medicine; Johns Hopkins University School of Medicine; Johns Hopkins School of Public Health

**Keywords:** Mycobacterium tuberculosis (MTb), Immunology Sex Differences, Antigen Presenting Cell (APC), Dendritic Cell (DC), DNA vaccine, MIP-3α (CCL20)

## Abstract

**Background:**

Tuberculosis (TB) remains a major cause of global morbidity and mortality. Efforts to control TB are hampered by the lengthy and cumbersome treatment required to eradicate the infection. Bacterial persistence during exposure to bactericidal antibiotics is at least partially mediated by the bacterial stringent response enzyme, Rel_Mtb_. A therapeutic DNA vaccine targeting Rel_Mtb_ has been shown to increase the efficacy of antitubercular drugs, and fusing macrophage-inflammatory protein 3α (MIP-3α), which interacts with CCR6 on immature dendritic cells (iDCs), to Rel_Mtb_ further increases the vaccine’s therapeutic efficacy. A secondary analysis of these prior studies elucidated prominent sex-based differences in vaccine therapeutic efficacy, with female mice showing improved microbial outcomes compared to males as a result of the Rel and MIP-3α-Rel vaccine constructs, with a greater sex-associated difference in the MIP-3α-Rel group. In the current study, we addressed the hypothesis that these sex-related differences are due to differential DC activation/function soon after vaccination.

**Methods:**

A EαGFP reporter vaccine model was used to track vaccine antigen presentation by an antibody Y-Ae which binds the Eα peptide tag in complex with I-A^b^ MHC-II molecules.

**Results:**

MIP-3α-EαGFP groups had more DCs presenting vaccine antigen infiltrating from the periphery, with more abundant Langerhans cells in males and greater CD8 + CD103 + cross-presenting dermal DCs in females. This model also shows there is greater DC activation, as measured by CD80 and MHC II MFI, by MIP-3α compared to EαGFP alone, especially in female mice.

**Conclusions:**

Our findings are consistent with the sex- and MIP-3α-related differences seen in the therapeutic model and supports the hypothesis that in both sexes MIP-3α enhances vaccine uptake and cell activation by peripheral iDCs. Additionally, Female mice showed greater levels of antigen presentation, especially in DCs able to cross-present antigen, explaining why they had the best outcomes. Further studies are required to understand underlying mechanisms and to link APC results directly to T-cell responses.

## PLAIN ENGLISH SUMMARY

Tuberculosis (TB) remains a major cause of global illness and death. Treating the bacteria that causes TB is di cult because some bacteria can shift into a dormant state that allows them to avoid killing by antibiotics. Our lab has developed a vaccine model that targets that dormant state using a unique enhancer of immune responses. Combination of antibiotics to kill the growing bacteria and our vaccine to kill the dormant bacteria has resulted in a significantly better treatment in animal models. After re-analyzing our previously published studies, we found something very interesting: female mice responded to vaccine treatment better than males, and this difference was more obvious in our vaccine including the immune enhancer. This leads us to believe the sex differences observed with our vaccine are due to differences in the specific immune cells that our enhancer targets.

To test this, we created a reporter vaccine that allows us to track which immune cells are interacting with our vaccine. We found that female mice interact with the vaccine more efficiently, that male and female mice rely on different cell subsets to interact with our vaccine, and that female mice favored cells that are known to be especially good for initiating T cell responses. We also found our immune enhancer increased activation of these immune cells, especially in female mice. These findings help explain why the female mice that received the vaccine responded better to the TB treatment. Further studies are required to more fully understand why this happens.

## BACKGROUND

*Mycobacterium tuberculosis* (Mtb) is the leading causative agent of tuberculosis (TB). Curative treatment for drug-susceptible TB requires a 6-month-long regimen of multiple antibiotics^1^ since a proportion of the bacteria can downshift their growth and metabolism, becoming tolerant to antibiotics.^2–5^ These so-called “persisters” show induction of the stringent response, which is regulated by the (p)ppGpp synthase/hydrolase, Rel_Mtb_.^6–8^

Our laboratory developed a therapeutic DNA vaccine that targets Rel_Mtb_ and which enhances the bactericidal activity of the first-line antitubercular drug, isoniazid (INH).^9^ We optimized the vaccine by fusing *rel*_*Mtb*_ to the gene encoding chemokine macrophage-inflammatory protein 3α (MIP-3α/CCL20), which interacts with the CCR6 receptor on immature dendritic cells (iDCs) and T-cells. This chemokine fusion has been shown in multiple studies to enhance the vaccine’s efficacy by specifically targeting vaccine antigen to iDCs.^10–17^ Our earlier studies found that there were significant sex-based differences in therapeutic efficacy of the Rel_Mtb_ vaccine (termed Rel), which were enhanced further by MIP-3α fusion (termed MIP-3α-Rel).

Sex-based differences in outcomes for infectious disease, autoimmunity, cancer, and vaccine efficacy have been widely observed.^18–20^ Antigen-presenting cells (APCs), which are essential for adaptive immune response initiation and especially critical in our vaccine model, have known differences in phenotype between females and males. In particular, APCs in females exhibit more efficient phagocytosis^21^ and antigen presentation^22^ under certain conditions. Dendritic cells, which are professional APCs, also demonstrate sex-based differences in immunity. In asthma models, female mice had elevated levels of DCs in the lungs and increased trafficking of DCs to the lymph nodes.^22–24^ Additionally, estrogens promote DC differentiation, competence, development, and functional responses.^25^

Since MIP-3α targets antigen to iDCs,^10–16^ we reasoned that the immune response at the earliest stages of antigen engagement and DC activity in the draining lymph nodes may be important contributors to the differences observed in vaccine efficacy between males and females. The central hypothesis of this study is females compared to males will show enhancement of DC functionality and antigen uptake and presentation *in vivo* post-vaccination that are more pronounced with MIP-3α-antigen fusion vaccines.

## METHODS

### Vaccines

Vaccine constructs utilizing the mammalian expression system pSecTag2b were prepared as naked plasmid in 1xPBS, as described in previous publications.^26^ The vaccine construct comprised of Eα peptide tag fused to eGFP sequnce (termed EαGFP) was constructed by Genscript (Piscataway, NJ) in a pUC plasmid with parallel design to the current, verified Rel vaccine, with HindIII and KasI sites on the 5’ end and BamHI on the 3’ end. The construct was cloned into our pSecTag2b vaccine plasmid by HindIII and BamHI sites to form the EαGFP vaccine. MIP-3α was cloned into the construct using HindIII and KasI to form the MIP-3α-EαGFP vaccine. Cloning was performed using standard laboratory protocols^26^, and clones were verified by restriction digest gel electrophoresis (Supplementary Fig. 1A–B) and by insert sequencing (JHMI Synthesis and Sequencing Facility [SSF]). Sequences are listed in Supplemental Data File 1.

EαGFP and MIP-3α-EαGFP DNA plasmids were maintained in DH5-α (Thermo Fisher Scientific, Waltham, MA) *E. coli*, grown in large cultures, and extracted utilizing Qiagen (Germantown, MD) endotoxin-free kits. Plasmids were verified for purity, supercoiling, and correctness by insert sequencing (Johns Hopkins Synthesis and Sequencing Facility), Nanodrop (Thermo Fisher Scientific, Waltham, MA) spectrophotometry, and agarose gel electrophoresis. Rel and MIP-3α-Rel vaccines have been used and verified previously.^26^

The EαGFP and MIP-3α-EαGFP vaccines were functionally validated by transfecting HEK293T-cells using Lipofectamine 3000 (Invitrogen, Waltham, MA) according to the manufacturer’s protocol. Cell lysates were analyzed by denaturing western blots with anti-C-myc primary (Biolegend, San Diego, CA), alkaline phosphotase-conjugated goat anti-mouse secondary antibody (Jackson Immunoresearch), and NBT-BCIP visualization reagent (Sigma Aldrich, St. Louis, MO), according to previously published protocols^27^ (Supplementary Fig. 1C). Cell media supernatant was concentrated 2x using Microsep Advanced 3K centrifugal filters (Pall Life Sciences, Port Washington, NY), analyzed by semi-denaturing blotting, and visualized for eGFP signal (excitation peak at 488nm, emission peak at 513nm) by a FluorChem Q camera (Alpha Innotech, San Leandro, CA) under Blue (475/42 nm) excitation and a green filter for detection (537/35 nm).

### Animal studies

**Figure 1:**
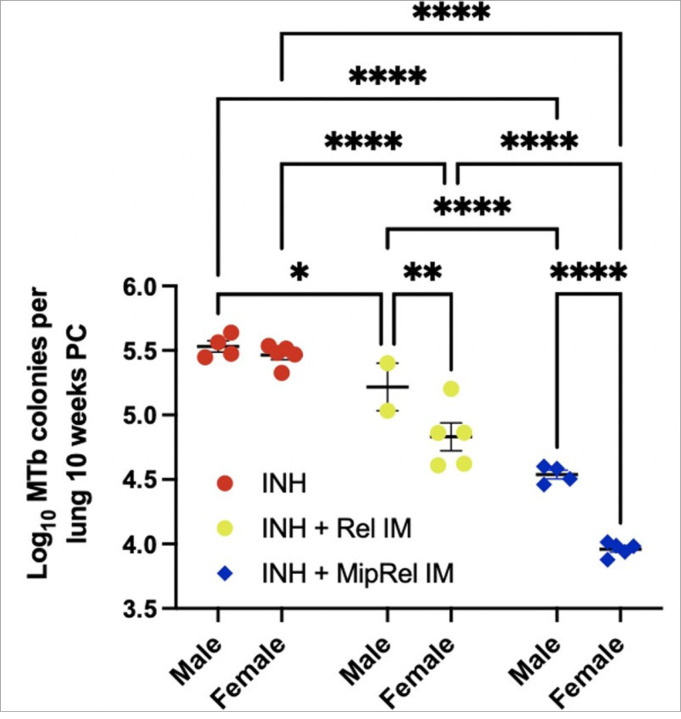
Sex differences of vaccine against Mycobacterium tuberculosis (MTb) stringent response protein, RelMTb. Differences in lung bacterial load 10 weeks post challenge of MTb infected mice receiving isoniazid (INH) alone, INH + Rel vaccine, and INH + MIP-3α-Rel vaccine from previously published dataset,^26^ newly stratified by sex of mice. *p<0.05, **p<0.01, ****p<0.0001

The methodology and experimental design for the study depicted in Fig. 1 have been detailed in a previous publication.^26^ Fig. 1 is a secondary analysis of the previously published dataset, stratifying select groups by sex. The remaining mouse studies utilized 6–8-week-old C57Bl/6 mice purchased from Charles River Laboratories (Wilmington, MA). Male and female mice were immunized with saline or 20 μg total of Rel, MIP-3α-Rel, EαGFP, or MIP-3α-EαGFP transfection-grade DNA plasmids injected as 10 μg in 50-μl doses into both mouse hind leg gastrocnemius muscles immediately followed with local electroporation using an ECM830 square wave electroporation system (BTX Harvard Apparatus, Holliston, MA). Each of the two-needle array electrodes delivers 15 pulses of 72V (a 20-ms pulse duration at 200-ms intervals).^12^ At 48 or 72 hours post-vaccination, mice were euthanized and draining popliteal nodes were harvested and processed. All mouse work was conducted using protocols approved by Johns Hopkins University IACUC.

### Flow Cytometry

Popliteal lymph nodes were harvested, and tissue was processed into single-cell suspensions according to laboratory protocols.^12,13^ Lymph node cells were harvested, processed, and stained on the same day to preserve cell viability. Cells were stained for markers of vaccine antigen protein and presentation (internal eGFP, Y-Ae), DC lineage (CD11c, CD11b, CD8α, B220, CD103, langerin, F4/80), DC trafficking and activation (CCR6, CCR7, CD80, CD86, MHC-II), exclusion of other immune cells (CD3, CD19), and Live/Dead. Antibodies were purchased from either Biolegend (San Diego, CA) or Invitrogen and details of clones, fluorophores, and dilution factors are in Supplemental Table 1. Stained cells were assayed using a Fortessa cytometer (Becton Dickinson, Franklin Lakes, NJ) in the SKCCC Flow Cytometry Technology Development Center (FCTDC). FlowJo (FlowJo, LLC Ashland, OR) software was used for analysis. Gates were determined using Full-Minus-One type staining controls. Since Y-Ae is known to have background signal in mature DCs without Eα^14^, Y-Ae gates were further adjusted based on control animals immunized with saline (vehicle), one per sex, to limit background. Gating structure of screening gates are outlined in Supplementary Fig. 2 and gates for specific APC populations are defined in Supplemental Figs. 3A–C.

### qRT-PCR

Mice immunized with Rel or MIP-3α-Rel vaccines had draining popliteal lymph nodes harvested at either day 2 or 3 post-immunization as noted in the figure legends, and the lymph nodes were flash-frozen in RNALater (Invitrogen, Waltham, MA) using a dry/ice + ethanol bath and stored at − 80° C. Lymph nodes were later thawed and homogenized, and RNA was immediately isolated using TRIzol reagent (Invitrogen, Waltham, MA) according to the manufacturer’s protocols. Day 3 nodal RNA was reverse-transcribed into cDNA by Qiagen’s RT^2^ First Strand Kit and then assayed using the RT^2^ Pro ler^™^ PCR Array: Mouse Dendritic & Antigen Presenting Cell, which requires RT^2^ SYBR Green ROX qPCR Mastermix. The qRT-PCR array was run on the StepOnePlus system (Applied Biosystems, Waltham, MA) in the Department of Molecular Microbiology and Immunology (MMI) Common Equipment Core. Day 2 nodal RNA was reverse-transcribed using SuperScript III First-Strand Synthesis System (Invitrogen, Waltham, MA) and assayed using TaqMan (Thermo Fisher Scientific, Waltham, MA) probes for Rac1 (Mm01201653 mh) and Clec4b2 (Mm02599731 m1) and TaqMan Fast Advanced Master Mix (Applied Biosystems, Waltham, MA) on MicroAmp Fast Optical 96-Well Reaction Plates (0.1mL) (Applied Biosystems, Waltham, MA). The qRT-PCR assays were run in triplicate using the manufacturer protocol on the Quant Studio 6 Pro Real-Time PCR System (Applied Biosystems, Waltham, MA) in the MMI Common Equipment Core.

### Statistics

For flow cytometry experiments, two independent experiments were performed for a total sample size of 7–8 mice per sex per group. For mean fluorescence intensity (MFI) analyses, to adjust for changes in fluorescent baselines across experiments, baseline measurements of MFI for each parameter (gated on immune cells, single cells, and alive cells) were calculated and averaged per group. Across-experiment shifts were calculated and multiplied to the values of the second experiment to normalize the dataset. Datapoints with < 2 cells were excluded from MFI analysis. For the qRT-PCR Array, 5 mice per group were utilized. Equal total RNA amounts from each mouse per sex/group were pooled to ensure adequate cDNA yield for successful array plate prep and qRT-PCR analysis. For the individual gene qRT-PCR, 4 mice were analyzed per group in triplicate. All the qRT-PCR results were analyzed as ΔCt normalized to a standard pool of housekeeping gene controls. All results comparing both group and sex were analyzed by two-way ANOVA with comparisons across rows and columns utilizing Fisher’s LSD Test. Data were stored in Microsoft Excel 2024 (Microsoft Corporation) (Supplemental Data File 2) and analyzed by Prism 10 (GraphPad Boston, MA). α = 0.05.

## RESULTS

### Secondary Analysis of Therapeutic Vaccine Study by Sex of Mice

A previous mouse Mtb challenge study compared the lung bacillary load 10 weeks post-treatment with each of the following regimens: INH alone; INH supplemented with Rel vaccine; and INH supplemented with MIP-3α-Rel vaccine.^26^ This study found that Rel vaccination increased the bactericidal activity of INH, and MIP-3α-Rel vaccination further increased its activity. Upon secondary analysis, it was clear that the therapeutic vaccines had a sex bias in their efficacy. Stratifying the data by sex, no difference in CFU was observed between the sexes in mice receiving INH alone. Females in the INH + Rel group had 57% fewer colony-forming units (CFU) compared to their male counterparts (p = 0.0039). For the INH + MIP-3α-Rel group, females had 74% fewer CFU compared to their male counterparts (p < 0.0001;Figure 1). Importantly, despite the sex bias, MIP-3α fusion enhanced the efficacy of the Rel vaccine in both sexes to varying degrees. These data showed the sex bias of our vaccine model and provided evidence that inclusion of MIP-3α in the vaccine construct enhanced the overall efficacy in both sexes but also heightened the female efficacy bias. Since MIP-3α is known to target CCR6 + iDCs and Langerhans cells,^10–16^ these observations led to our hypothesis that sex differences in APC function, specifically differences in targeting vaccine antigen to DCs, may be contributing to the observed female bias.

### EαGFP and MIP-3α-EαGFP Model System and Validation

**Figure 2:**
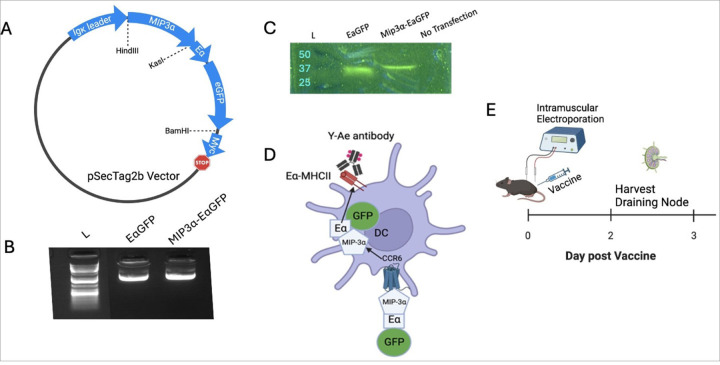
MIP-3α-EαGFP Model. A) Map of expressed region of vaccine plasmid with regions and cloning sites labeled. B) Confirmations of transfection-grade plasmid preps to show DNA purity and supercoiling C) EαGFP and MIP-3α-EαGFP vaccines verified in vitro by transfection of HEK293T-cells. Cell media was analyzed by semi-denaturing gel followed by blotting and GFP visualization under UV excitation and a green filter D) Model of system. Secreted vaccine protein will be targeted to iDCs via interaction between MIP-3α and CCR6. Internalization can be tracked by GFP and antigen presentation by Y-Ae antibody designed to interact with I-Ab MHC-II loaded with Eα peptide. E) Schedule of manuscript experiments: Vaccines administered into mouse gastrocnemius muscle with electroporation. Draining popliteal nodes were harvested two to three days later. Illustrations made using Biorender.com

To detect and track which cells take up and present vaccine antigen, our laboratory created EαGFP and MIP-3α-EαGFP reporter vaccines (Fig. 2A), with verified plasmid purity (Fig. 2B) and *in vitro* transfection ability (Fig. 2C). The EαGFP vaccine functions as a DC reporter^28,29^ by providing two mechanisms for tracking: GFP as a visible cue for phagocytosed protein and the Eα peptide, which has a known antibody (Y-Ae) that binds to the processed peptide in complex with I-A^b^ MHC-II on C57Bl/6 mouse APCs, allowing for identification of processed vaccine antigen (Fig. 2D). Mice were vaccinated with the EαGFP or MIP-3α-EαGFP reporter vaccine and at 48 and 72 hours post-vaccination, popliteal nodes were harvested, processed into single-cell suspensions, and analyzed by flow cytometry (Fig. 2E).

### Sex and MIP-3α Differences in Proportion of APC’s Presenting Antigen

**Figure 3:**
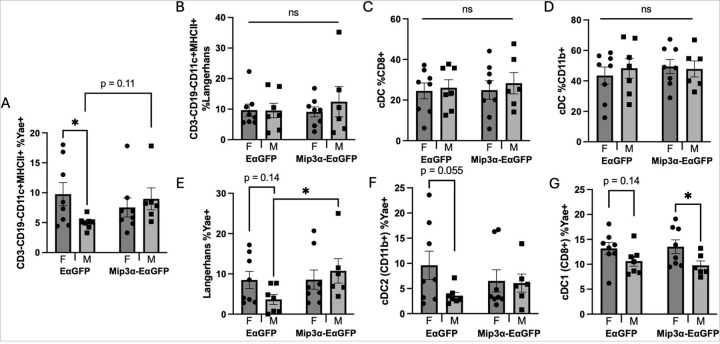
Sex and MIP-3α differences of antigen presentation. A) Antigen presenting cells (APCs) as defined by CD3-CD19-CD11c+MHCII+ shown as percentage positive for Y-Ae signal. Panels B-D show overall lymph node populations of APCs, including Langerhans cells (B; CD207+F4/80-), and cDCs (CD207-F4/80-) divided into CD8+ (C) and CD11b+ (D) populations. Panels E-G de ne the Y-Ae+ proportions of those APC subsets. Data are representative of two independent experiments, n=6–8. *p<0.05, ns = not significant, nonstatistically significant p-values labeled.

An initial hypothesis was that MIP-3α was enhancing overall vaccine antigen presentation, which is known to have a female bias in other model systems.^22,25^ In Fig. 3, both sex and vaccine type differences are shown in APC subtype and the surface distribution of Y-Ae-labeled vaccine antigen distribution. The gating strategy is shown in Supplemental Figs. 2–3. All presented data were selected for immune cells by forward scatter (FSC) vs side scatter (SSC), single cells, alive cells, CD3-, CD19-, CD11c+, and MHC-II+, defining APCs generally, and all cells passing through specific subtype gates previously had passed through the APC gates. There is a significant sex difference in the percentages of Y-Ae + APCs in the EαGFP group, with females averaging about twice that of males (p = 0.042). However, the MIP-3α-containing vaccine surprisingly showed similar APC presentation levels in both sexes. When comparing the same sex across the vaccine types, the results show a nonstatistically significant difference in males vaccinated with the construct containing MIP-3α (mean 74.2% higher Y-Ae signal; p = 0.11) relative to those vaccinated only with EαGFP, and a similar Y-Ae signal in both female vaccinated groups (Fig. 3A). Overall, APC antigen presentation in the standard vaccine model showed a significant female bias, as hypothesized, but the MIP-3α-containing vaccine clearly displayed different dynamics. We next analyzed the overall cell infiltrate to see if MIP-3α immunized animals had greater overall APC trafficking to the popliteal lymph nodes. When comparing APC subtypes (Figs. 3B–D), there are no significant differences in sex or vaccination type in the percentage of Langerhans cells (Langerin+, F4/80+), type 1 conventional DCs (cDC1s; Langerin-, F4/80-, CD8+), or type 2 conventional DCs (cDC2s; Langerin-, F4/80-, CD11b+). There were also no significant differences in the percentage of alive T-cells, B-cells, or GFP^+^ cells by sex or vaccine type that could confound the results (Supplementary Fig. 4). From these data, we concluded that the overall cell composition of the node remained consistent independent of vaccine formulation and sex.

Next, to test the hypothesis that there may be differences in antigen uptake across the specific APC subtypes, we analyzed which cells were positive for reporter vaccine antigen. Among the groups receiving the EαGFP construct, the mean percentage of Y-Ae + Langerhans cells was 2.32 times higher in females than in males (p = 0.14), and there was no difference in this cell type the MIP-3α-EαGFP group. Comparing within the same sex across vaccination groups, there was a significant difference in males receiving MIP-3α-EαGFP, as this group had a mean that was 2.94 times higher that of males receiving Eα-GFP (p = 0.045; Fig. 3E). This indicates that in males MIP-3α targets Langerhans cells to be a significant APC to infiltrate from the periphery, take up vaccine antigen, and travel to the lymph node. Next, studying the percentage of cDC1 (CD8+) DCs that are Y-Ae+, we found a nonstatistically significant sex difference in the EαGFP group, with females averaging 23.8% higher than males (p = 0.14) and a significant sex difference in the MIP-3α-EαGFP group, with females averaging 38.5% higher than males (p = 0.04; Fig. 3F). This provides evidence that in the MIP-3α-EαGFP group there is a female bias for cDC1s to uptake vaccine antigen and present it to T-cells in the lymph node. Notably, though, there is not a difference in the same sex analyses across vaccine types, so these data can help explain the sex difference but not the MIP-3α enhancement. Finally, examining the percentage of Y-Ae + cDC2 (CD11b+) DCs, we found a nearly significant sex difference in the EαGFP group, with females averaging 2.68 times higher than males (p = 0.055) and no difference in the MIP-3α-EαGFP group (Fig. 3G). Analyses within the same sex across vaccine groups showed no significant differences. Overall, the APC subtypes (Fig. 3E–G) mimic the antigen presentation data (Fig. 3A) for the EαGFP vaccine, with females being nonstatistically significantly higher in each. For the MIP-3α-EαGFP vaccine, only the cDC1 population is showing a female bias in presentation. Interestingly, the only differences found across vaccination groups within the same sex was an increase in antigen presentation in Langerhans cells in male mice. These data support our theory that the sex differences observed in response to vaccination is in part due to different preferences for APC subtypes.

### Sex and MIP-3α Differences in APC Activation Levels

In addition to studying the *ex vivo* antigen presentation proportions across APC subtypes, we also examined activation markers of the APCs presenting Y-Ae to determine if there were sex differences or MIP-3α differences as a result of vaccination. Since MIP-3α enhances vaccine efficacy and increases endpoint T-cell responses, ^10–16^ which could not be fully explained by increased numbers of antigen loaded-APCs in the draining node (Fig. 3), we hypothesized that MIP-3α could increase DC activation, potentially leading to a more robust T-cell response. Therefore, we examined the differences in expression of CD80 and MHC-II in Langerhans cells, cDC2 (CD11b+) DCs and cDC1 (CD8+) DCs.

**Figure 4:**
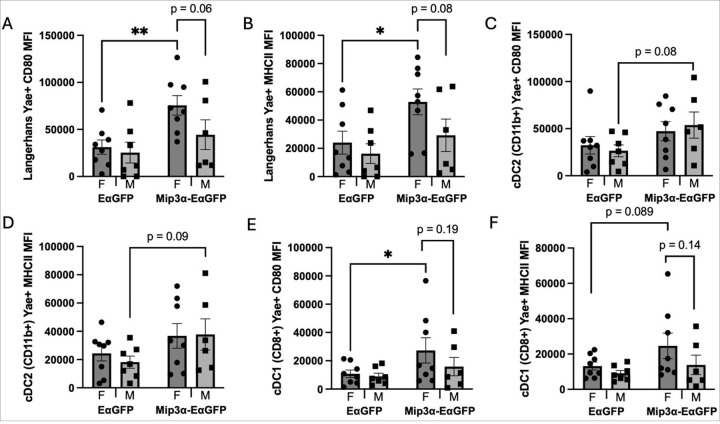
Sex and MIP-3α differences of APC activation within Y-Ae positive populations. Langerhans (A-B), CD11b+ cDC (C-D), and CD8+ cDC (E-F) cell populations were analyzed for surface expression levels of CD80 (A, C, E) and MHCII (B, D, F) by comparing mean fluorescence intensity (MFI) measures. Data are representative of two independent experiments, n=6–8. *p<0.05, **p<0.01, nonstatistically significant p-values labeled.

We found that female recipients of the MIP-3α vaccine construct had a 2.42-fold higher CD80 surface abundance on Y-Ae + Langerhans cells compared to female recipients of EαGFP (p = 0.0061; Fig. 4A). Relative to their male counterparts, female mice receiving MIP-3α-EαGFP had 70.4% higher CD80 surface abundance on Y-Ae + Langerhans cells (p = 0.06). These findings are also consistent with the MHCII MFI data, as a significant, 2.2-fold increase was observed in the females receiving MIP-3α-EαGFP vs. EαGFP (p = 0.023; Fig. 4B). A nonsignificant difference in MHCII surface abundance was observed between sexes in the MIP-3α-EαGFP group, with females averaging 81.2% higher than males (p = 0.08). These findings our consistent with our hypothesis that MIP-3α enhances activation of Langerhans cells. Interestingly, there is a female bias in the MIP-3α group for CD80 and MHC II surface abundance.

Next, studying cDC2 (CD11b+) DCs, we found a nonstatistically significant MIP-3α difference in the CD80 surface abundance on cDC2 (CD11b+) cells that are Y-Ae + with MIP-3α-EαGFP males averaging 2 times higher than EαGFP males (p = 0.08; Fig. 4C). Similarly, there is a nonstatistically significant MIP-3α difference in the MHC II surface abundance on cDC2 (CD11b+) cells that are Y-Ae + with MIP-3α-EαGFP males averaging 2.1 times higher than EαGFP males (p = 0.09; Fig. 4D). This again shows that MIP-3α is able to enhance activation marker levels. Within the cDC2 subset there are no significant differences in sex.

Finally, examining cDC1 (CD8+) DCs, there is a significant MIP-3α difference in the CD80 surface abundance on cDC1 (CD8) cells that are YA-e + with MIP-3α-EαGFP females averaging 2.5 times higher than EαGFP females (p = 0.049). There is also a nonstatistically significant sex difference in the MIP-3α-EαGFP group with females averaging 72.6% higher than males (p = 0.19; Fig. 4E). We observe a nonstatistically significant MIP-3α difference in the MHC II surface abundance of cDC1 (CD8) cells that are YA-e + with MIP-3α-EαGFP females averaging 86.7% higher than EaGFP females (p = 0.089). A nonstatistically significant sex difference is also present in the MIP-3α-EαGFP group with females averaging 77.9% higher than males (p = 0.14; Fig. 4F). Again, this shows that MIP-3α is able to enhance activation marker surface levels.

Overall, the findings in Fig. 4 support the hypothesis that the enhanced adjunctive bactericidal activity afforded by the MIP-3α fusion (Fig. 1) is likely not due to enhanced antigen presentation and T-cell activation, but, at least in part, due to enhanced activation of APCs, which are better able to productively present antigen to T-cells. Further, expression of APC activation markers was most robust in female mice, potentially explaining the observed sex bias of the therapeutic efficacy of MIP-3α-Rel.

### Cross-Presenting CD8 + CD103 + DCs

**Figure 5:**
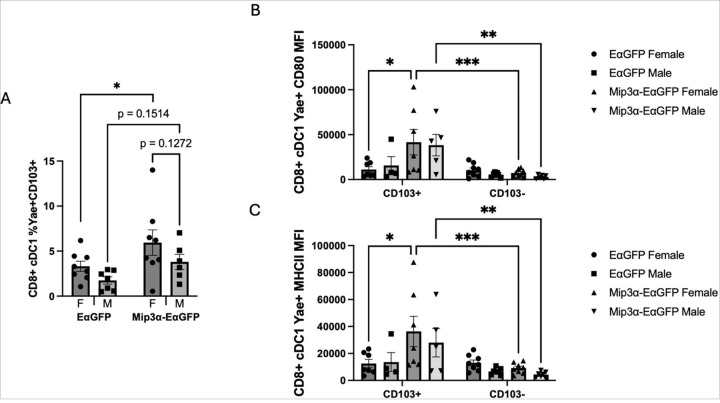
Sex and MIP-3α differences of the CD8+ cDC population positive for both Y-Ae and CD103. A) Proportion of CD8+ cDC cells positive for both Y-Ae and CD103. B) CD80 and C) MHCII MFI measurements of the CD8+ cDC population positive for Y-Ae stratified by CD103 positivity. Data are representative of two independent experiments, n=6–8. *p<0.05, **p<0.01, *** p<0.001, nonstatistically significant p-values labeled

We found that cDC1 DCs consistently have a female bias, both in vaccine antigen presentation and cell surface activation marker levels, which led us to investigate if these cells were lymphoid resident (CD103-) or trafficking from the vaccine site (CD103+). We found that MIP-3α enhances the number of Y-Ae + and CD103 + positive cells, with MIP-3α-EαGFP females averaging 78.9% higher than EαGFP females (p = 0.046) and MIP-3α-EαGFP males averaging 2.2 times higher than EαGFP males (p = 0.15) (Fig. 5A). There is also a nonstatistically significant sex difference in the MIP-3α-EαGFP group with females averaging 56.3% higher than males (p = 0.13). This provides evidence that a greater proportion of the Y-Ae + cDC1 DCs observed in Fig. 3F are from the CD8 + CD103 + subtype in the MIP-3α-EαGFP group as compared to the EαGFP group, while maintaining similar sex difference proportions.

We then stratified the activation signal data from the cDC1 Y-Ae + population by CD103 positivity. CD80 (Fig. 5B) and MHCII (Fig. 5C) MFI levels show a clear phenotype: the majority of the enhanced activation signal observed in cDC1 cells (Figs. 4E–F) are attributable to the CD8 + CD103 + subset, which are almost exclusively present in the MIP-3α-EαGFP groups. A comparison of the activation levels across the vaccine groups within each sex revealed that MIP-3α-EαGFP females had significantly higher CD80 (3.7x; p = 0.011) and MHCII (2.9x; p = 0.014) MFI than EαGFP females. Although males showed a similar phenotype, no statistical difference was observed, in large part due to a loss of statistical power from several samples in the EαGFP males being excluded due to too few CD103 + cDC1s to analyze by MFI. Interestingly, activation levels did not show any sex bias. These findings in part explain why female mice receiving MIP-3α-EαGFP had the most robust response to therapy (Fig. 1), since they had the highest proportions of CD8 + CD103 + DCs (Fig. 5A), which are highly activated by MIP-3α (Figs. 5B and C).

### Analysis of Total Lymphoid RNA

**Figure 6:**
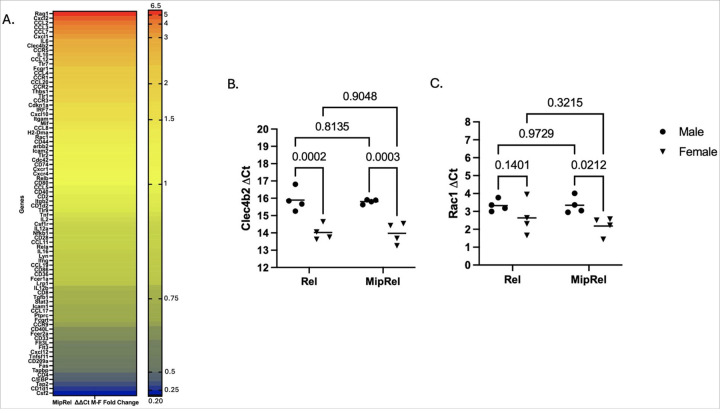
(A) Heatmap of the Day 3 Mouse Dendritic & Antigen Presenting Cell qRT-PCR Array presenting the fold change of the ΔΔCt gene expression comparing MIP-3α-Rel females to MIP-3α-Rel males. (B) Day 2 Clec4b2 ΔCt for Males and Females comparing the Rel and MIP-3α-Rel vaccine. (C) Day 2 Rac1 ΔCt for Males and Females comparing the Rel and MIP-3α-Rel vaccine. *p<0.05, ***p<0.001, ns = not significant.

To determine which genes might be contributing to the sex difference phenotype, RNA was isolated from mouse popliteal nodes 3 days post-vaccination and processed for the RT-qPCR array. Figure 6A outlines the ΔΔCt fold difference between female and male mice after MIP-3α-Rel vaccination (Male ΔCt – Female ΔCt). Females were found to express Rag1, CCL12, CCL2, CCL20, CCL3, CCL4, CCL7, CCR1, CCR5, CXCL1, Clec4b2, CXCL2, FCGR1, IL-10, IL-6, and TLR7 at a > 2-fold higher level than males. Males expressed CD1d1, CD209a, CD4, C/EPB, CSF2, Fas, TAP2, and TAPBP at a nearly 2-fold higher level than females. Based on the sex difference data from the RT-qPCR array, the previous flow data, and the literature, Rac1 was selected to repeat qRT-PCR analysis due to its effects on DC motility,^30^ which could impact DC infiltration at the site of vaccination. Clec4b2 was also selected to analyze as it impacts CD8 + T-cell cross-presenting,^31^ which is highly prevalent in cDC1 DC subtypes.^32^ Fig. 6B and C outline the sex and MIP-3α differences in ΔCt values for these genes. We observed a significant difference in Clec4b2 ΔCt between the sexes for the Rel vaccinated mice (p = 0.0002) and the MIP-3α-Rel vaccinated mice (p = 0.0003), with males averaging about 13% higher than females in both groups. There was no significant or nonstatistically significant difference between the Rel vaccine group and the MIP-3α-Rel vaccine group (Fig. 6B). This supports our findings that female mice have greater numbers of cDC1 DCs that can cross-present antigen. We also found there is a significant difference in Rac1 ΔCt between the sexes for the MIP-3α-Rel vaccine group, with males averaging 53% higher than females (p = 0.021). A nonstatistically significant sex difference is also present in the Rel group, with males averaging 26.6% higher than females (p = 0.14; Fig. 6C). This also indicates that females may have increased motility, leading to more infiltrating cDC1 DCs at the site of vaccination.

## DISCUSSION

Utilizing the EαGFP reporter vaccine is a powerful tool to characterize the APCs that are actively taking up and presenting vaccine antigen, including the effects vaccine formulations and sex have on these APCs in the days following vaccination. As described in Fig. 2D, the Eα peptide, when presented by I-A^b^ MHC-II molecules in C57BL/6 mice, can be analyzed with the antibody Y-Ae in flow cytometric assays to determine Eα presentation for different APC types. Based on previous results in *in vitro* DC models with MIP-3α fusion vaccines, it has been shown that targeting with CCR6 was able to induce successful cross-presentation of antigens into both MHC-I and MHC-II pathways.^14^ However, this study is the first to analyze the effects of MIP-3α on APCs *ex vivo* to assay the real time antigen presentation capabilities of different types of APCs post-vaccination. This is also the first study to assess how MIP-3α fusion to a vaccine antigen affects differences in the sexes in regard to both microbiological endpoints and early-stage APC activity. Additionally, this is the first study to our knowledge to assess sex differences in vaccine uptake utilizing the Eα peptide – Y-Ae antibody tracking system.

In the literature, females have shown to have overall more active immune responses compared to males. Specifically , female APCs are more efficient in phagocytosis, antigen presentation, and trafficking to the lymph node than males.^21–23^ Studies have also found that estrogens can enhance DC development and function,^25^ and estradiol can increase IL-6 and MCP-1 (CCL2) production by iDCs, enhance DC migration towards chemokines in the lymph node, and increase DC ability to stimulate T-cells without fundamentally altering the DCs.^33^ Based on previous studies, we hypothesized that females would show greater levels of antigen presentation and that MIP-3α presence in the vaccine would enhance presentation in DCs coming from the vaccination site and able to cross-present antigens. Overall, our data support this hypothesis with some further intriguing findings.

When comparing sex and MIP-3α differences for vaccine antigen uptake in different DC subsets, we found that MIP-3α increased Y-Ae + DC infiltration from the periphery, with MIP-3α males having the highest percentage of Langerhans cells and MIP-3α females the highest percentage of CD8 + CD103 + dermal DCs (Figs. 3 and 5). We also observed no differences in general numbers of APCs, T-cells, B-cells or GFP by sex or vaccine type that could confound the results (Fig. 3B–D; Supplementary Fig. 4). CD8 + CD103-DCs are non-migratory cells that reside in the lymph node^34^ and CD8 + CD103 + are dermal DCs with high migratory potential that, when presented with antigen, travel to the draining lymph node.^35^ Both cDC1 subtypes (CD8 + and CD8 + CD103+) are known to effectively cross-present antigen to CD8 + T-cells upon activation.^32^ Further, CD8 + CD103 + DCs have been found to be essential in the phenomenon of DC cross-dressing, which is especially important in DNA vaccinations.^32^ In the literature, there are known sex differences in these DC subsets and in CD8 + T-cell activation. A recent study found that DCs from female mice were better at taking up FITC injected into the skin than male counterparts,^36^ which is consistent with our antigen presentation data. Additionally, while male mice typically have increased numbers of CD8 + T-cells, female mice have higher numbers of activated CD8 + T-cells in different models,^18,37–39^ which could result from increased cross presentation from cDC1 (CD8 + and CD8 + CD103+) DCs. Previous studies in an asthma model have also found that MHC II expression and CD103 + DC antigen uptake was higher in females than males^23^, providing evidence for why females have more cDC1 (CD8 + CD103+) Y-Ae + DCs compared to males. These factors contribute to increased CD8 + T-cell cross-presentation/dressing and activation, and a more favorable overall immune response in female mice.

In addition to sex differences, we also saw MIP-3α differences in activation due to vaccination (Fig. 4). In Y-Ae + Langerhans cells, cDC1s (CD8+), and cDC2s we found MIP-3α groups had the highest CD80 and MHC II surface expression when compared to the EαGFP groups. These data indicate that regardless of APC subtype, mice immunized with MIP-3α-fusion vaccines have APCs with greater surface levels of activation markers. We also observed a female sex bias in activation in the Langerhans cells and cDC1 (CD8+) DCs. A majority of the CD80 and MHC II surface expression observed in the cDC1 DCs was from CD8 + CD103 + DCs, which are greatly increased in animals vaccinated with the MIP-3α-fusion vaccine (Fig. 5). Another study found that female mice had more mature CD103 + cDC1 cells in the skin than male mice, ^36^ which could be contributing to why we may see more nodal trafficking and activation by this subtype. Further details into the mechanisms of activation, cross-presenting or dressing, and nodal trafficking will be the focus of future studies.

These findings are further supported by our qRT-PCR panel in Fig. 6A. Many of the genes upregulated in MIP-3α females are chemokines involved in immune cell motility and attracting cells to the node, such as CCL12, CCL2, CCL3, CCL7, CCR1, CCR5, and CXCL1.^17,40^ Fig. 6C shows that females have enhanced Rac1 expression, nonstatistically significant in the Rel group and significant in the MIP-3α-Rel group. Rac1 is a Rho GTPase that has many functions including actin cytoskeleton rearrangement^30^ which may lead to increased DC motility. This is also consistent with the literature as studies have found that estradiol is essential for DC migration.^33^ Increased cell motility in females could result in more periphery DCs moving to the site of antigen exposure to process and uptake vaccine antigen. High levels of chemokines could also increase dermal DC migration to bring vaccine antigen to the draining lymph nodes, contributing to more CD8 + T-cell cross-presentation and resulting in an improved immune response to vaccination.

Figure 6A also highlights that many of the genes upregulated in female mice receiving the MIP-3α construct are associated with CD8 + T-cell recruitment and cross-presentation. In the literature, CCR6/CCL20 recruits DCs that are responsible for CD8 + T-cell cross-presenting^41^ and CCL3 can enhance CD8 + T-cell infiltration through CD103 + DC recruitment.^42^ Studies have also shown after vaccination, naïve CD8 + T-cells in the draining lymph node secrete CCR5 which recruits immune cells that secrete CCL3 and CCL4, resulting in increased memory CD8 + T-cells in the nodes.^43^ This is further supported in Fig. 6B where Clec4b2, or DCAR1, is significantly expressed more in females than males in both groups. Clec4b2 is found on the subset of cDC1 (CD8+) DCs and is involved in T-cell cross-presenting.^31^ Coupled with the increased levels of Y-Ae + cDC1(CD8 + and CD8 + CD103+) DCs, these genes could also contribute to the enhanced cross-presenting of CD8 + T-cells, thus enhanced immune response in females. More detailed analysis of T-cell response induction will be the subject of future work.

The genes that are upregulated in males receiving the MIP-3α construct include CD209a (DC-SIGN), especially important in monocyte-derived DC-driven T-cell responses,^44^ and multiple genes that signal or induce DC maturation, such as C/EPB, Fas, and Tap2.^45–47^ This provides further evidence that MIP-3α is associated with sex biases in terms of which APC types are targeted, and further studies will need to confirm the role of monocyte-derived DCs in this system. These data also suggest the female APCs likely matured before this time point, considering the higher surface levels of activation markers observed in the female MIP-3α group (Figs. 4 and 5) combined with this genetic shift towards males with maturation gene expression (Fig. 6A). The location and temporal dynamics of cellular activation will also be analyzed in greater detail in future studies.

## CONCLUSIONS

Overall, our studies show that therapeutic vaccination with the *M. tuberculosis* antigen Rel_Mtb_ enhances the bacterial clearance by antibiotics with a female bias, and that fusion of the antigen to MIP-3α enhances both the overall response and the sex bias. The data presented here support the hypotheses that fusing MIP-3α to the antigen leads to better targeting of APCs at the vaccination site, such as Langerhans and CD103 + dermal DCs, and that female APCs have better antigen presentation capacity than males, including cross-presenting DCs. Interestingly, we found that the APCs from mice immunized with MIP-3α-fusion led to increased APC activation. We hypothesize the enhancement of therapeutic efficacy is in part due to the intriguing increase in APC activation markers primarily in the MIP-3α-antigen immunized mice combined with a female bias of cDC1 (CD8 + and CD8 + CD103+) DC vaccine antigen presentation, resulting in highly activated DCs that can cross-present to CD8 + T-cells, prompting them to elicit a strong immune response. Future investigations will also include the type 2 innate lymphoid cell (ILC2) population as a recent study suggests they play a major role in sex differences regarding DC accumulation and activation and are negatively regulated by androgens.^36^ More research studying the mechanisms involved in the sex differences observed as a result of vaccination, such as direct and indirect hormonal influences as well as sex chromosome influences, is pivotal to fully understanding how vaccination elicits a more robust immune response, specifically in females, and how to best target the stringent response.

## Supplementary Material

Supplement 1

## Data Availability

Presented data are provided in the submission supplement. Any other data or materials are available upon reasonable request.

## HIGHLIGHTS

- Targeting the stringent response pathway of MTb with a therapeutic vaccine to the Rel_Mtb_ protein combined with an antibiotic provided strong anti-tuberculin efficacy that had a female bias. Directing the vaccine to immature dendritic cells by fusing to MIP-3α enhanced the treatment efficacy overall as well as the female bias.
- Utilizing an EαGFP reporter vaccine model to track vaccine antigen presentation, female mice had higher overall antigen presentation rates.
- MIP-3α-EαGFP groups, and especially the females, showed greater DC activation as measured by CD80 and MHC II surface expression.
- When comparing MIP-3α-EαGFP groups to EαGFP groups, male mice showed higher rates of presentation with Langerhans cells and females showed higher rates of presentation with CD8+CD103+ cross-presenting dermal cDC1s that are strongly activated.
- Genes related to cell trafficking and cross presentation have female biases in the lymph node.
