# Supplementary material for: MIP-3α-antigen fusion DNA vaccine enhances sex differences in tuberculosis model and alters dendritic cell activity early post vaccination
Source: Res Sq. 2025 Jan 14:rs.3.rs-5663995. Preprint. [Version 1] doi: 10.21203/rs.3.rs-5663995/v1 (PMC11774437; doi:10.21203/rs.3.rs-5663995/v1)
Supplement: Supplement 1 [file NIHPPRS5663995v1-supplement-1.pdf]

Supplementary Files

This is a list of supplementary files associated with this preprint. Click to download.

- Supplementalfileslegends.docx
- FigS1.jpeg
- FigS2.jpeg
- FigS3.jpeg
- FigS4.jpeg
- SuppData1vaccinesequences.pdf
- SuppData2Dataset121724.xlsx
- TableS1.jpeg
